# A Single-Phase Embedded Z-Source DC-AC Inverter

**DOI:** 10.1155/2014/539297

**Published:** 2014-07-16

**Authors:** Se-Jin Kim, Young-Cheol Lim

**Affiliations:** Department of Electrical Engineering, Chonnam National University, Gwangju 500-757, Republic of Korea

## Abstract

In the conventional DC-AC inverter consisting of two DC-DC converters with unipolar output capacitors, the output capacitor voltages of the DC-DC converters must be higher than the DC input voltage. To overcome this weakness, this paper proposes a single-phase DC-AC inverter consisting of two embedded Z-source converters with bipolar output capacitors. The proposed inverter is composed of two embedded Z-source converters with a common DC source and output AC load. Though the output capacitor voltages of the converters are relatively low compared to those of a conventional inverter, an equivalent level of AC output voltages can be obtained. Moreover, by controlling the output capacitor voltages asymmetrically, the AC output voltage of the proposed inverter can be higher than the DC input voltage. To verify the validity of the proposed inverter, experiments were performed with a DC source voltage of 38 V. By controlling the output capacitor voltages of the converters symmetrically or asymmetrically, the proposed inverter can produce sinusoidal AC output voltages. The experiments show that efficiencies of up to 95% and 97% can be achieved with the proposed inverter using symmetric and asymmetric control, respectively.

## 1. Introduction

In general, a single-phase DC-AC inverter is a full-bridge inverter composed of four switches and a method using two converters with bipolar output voltages. A full-bridge inverter has weaknesses in that the AC output voltage is limited to below the input voltage, and a boost converter must be used to generate an output voltage over input voltage. A single-phase DC-AC inverter [1] using two DC-DC converters (Buck, Boost, Buck-boost, etc.) generates AC output voltages by the difference of the output capacitor voltages of each converter. This kind of inverter is advantageous because it does not require an LC output filter and their AC output voltage levels are not limited by the input voltages. However, this method has a disadvantage in that the voltage stress of the entire system increases because the output capacitor voltage of the DC-DC converter is only unipolar on the off-set voltage over the input voltage. Recently, embedded Z-source DC-AC converters [2–5] with bipolar output capacitor voltage have been proposed.

There is no need to consider off-set voltage, and direct AC voltage output can be obtained from each converter. Because the positive half-cycle of the AC output voltage from one embedded Z-source converter is limited by the input voltage, it is the same as a single bridge inverter. But, the output of the Buck or Boost AC voltage can be in the negative half-cycle voltage of the embedded Z-source converter, regardless of the input voltage. Therefore, a single embedded Z-source DC-AC inverter that combines the asymmetrical output voltage from two embedded Z-source converters is proposed.

In the proposed method, the output of a single AC voltage that is the same as the input voltage level is possible with an output voltage of the converter that is lower than that of a DC-AC inverter composed of a conventional DC-DC converter. And the proposed inverter can implement the same performance as the two-stage single phase full-bridge inverter using a boost converter. To verify the validity of the proposed system, an experiment was performed with a DC source voltage of 38 V. A DSP (TMS320F28335)-based single-phase embedded Z-source DC-AC inverter was used for the experiment.

## 2. The Proposed Inverter

### 2.1. The Proposed Embedded Z-Source Inverter

**Figure 1:**
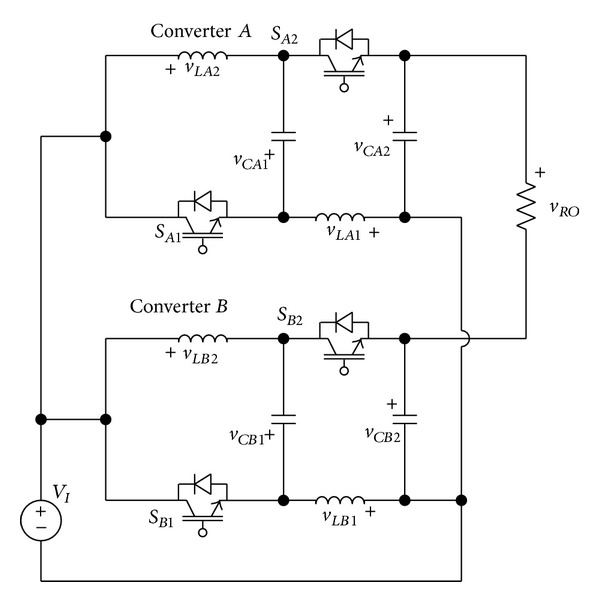
The proposed embedded Z-source DC-AC inverter.

Figure 1 shows the proposed single-phase embedded Z-source DC-AC inverter that consists of two embedded Z-source DC-DC converters.

**Figure 2:**
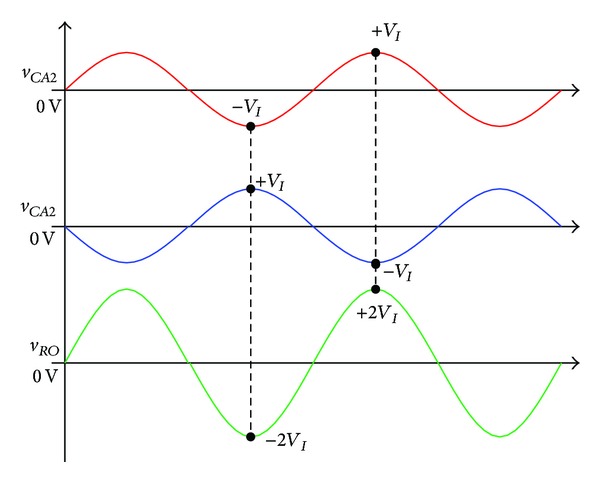
Output voltage principle of the proposed single-phase embedded Z-source DC-AC inverter.

The proposed inverter consists of common DC power source (*V*_*I*_), two embedded Z-source converters (Converter *A*, Converter *B*), and common AC load (*R*_*O*_). The output AC voltage (*v*_*RO*_) of the proposed inverter is the difference between the output voltage (*C*_*A*2_) of converter *A* and the output voltage (*C*_*B*2_) of converter *B*, as shown in Figure 2. Consequently, it is possible for the embedded Z-source DC-AC inverter to generate more than double the voltage of one converter output voltage [2].

As shown in Figure 2, converters *A* and *B* generate the output voltages with a 180° phase difference, and the output voltage of each converter is expressed by

(1) vCA1=−VI,vCA2=2D−1DVI=VCA2sin⁡ωt,

(2) vCB1=−VI,vCB2=1−2D1−DVI=VCB2sin(ωt+π),

where *D* denotes the turn-on time during one cycle time *T*_*s*_  of switching for each converter, and the time ratio of the turn-off time is (1 − *D*). In other words, *D* is the duty ratio of the embedded Z-source DC-AC inverter. The output voltage of proposed inverter is given by

(3) $$\begin{array}{c} v_{RO}=v_{CA2}-v_{CB2}=\frac{\left(2D-1\right)V_{I}}{D\left(1-D\right)}\sin\omega t. \end{array}$$

**Figure 3:**
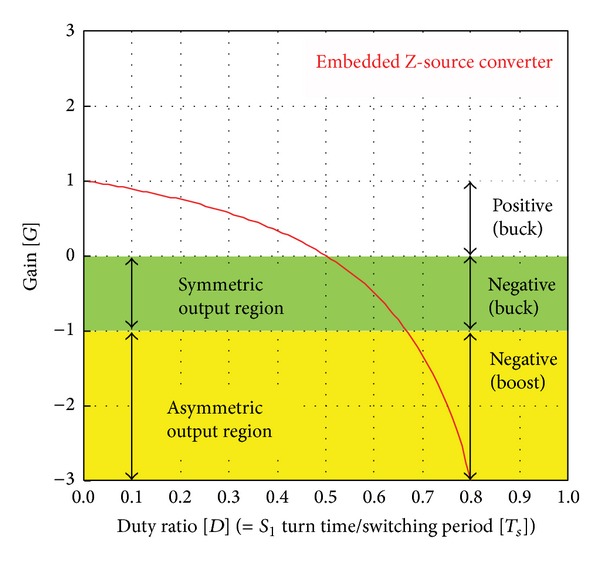
Gain curve of embedded Z-source DC-DC converter *B*.

The output area of embedded Z-source converters *A* and *B* can be verified by (1).  Figure 3 shows the gain curve of the embedded Z-source converter. Because the output capacitor voltage of the converter *A* is equal to the DC input voltage *V*_*I*_ under the condition that *D* = 1.0, Buck mode is performed only in the positive half-cycle voltage.

The output voltage of the converter is 0 V at *D* = 0.5, and the negative half-cycle voltage for the DC input voltage is theoretically limitless voltage gain (*G*). The output voltage of converter *B* has a 180° phase difference for the capacitor voltage of converter *A*, but *D* (the duty ratio) and *G* (the gain) are equal to those of converter *A*.

### 2.2. The Method for the Asymmetrical Voltage Control

As shown in Figure 5, *T* − *W*(+*V*_*I*_ ~ −*V*_*I*_) and *S* − *X*(+*V*_*I*_ ~ −*V*_*I*_) are defined as the symmetrical output cycle, which is the period in which it is possible to output the same magnitude as the maximum value of |*V*_*I*_| in the negative half-cycle of the embedded Z-source converter.

**Figure 4:**
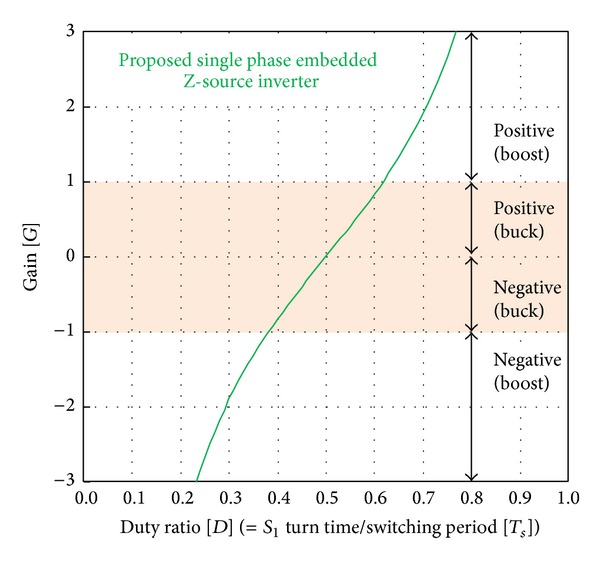
Gain curve of the proposed single-phase embedded Z-source DC-AC inverter.

The higher negative half-cycle *U* − *W*(+*V*_*I*_ ~ −2*V*_*I*_), *V* − *W*(+*V*_*I*_ ~ −3*V*_*I*_), *S* − *Y*(+*V*_*I*_ ~ −2*V*_*I*_), *S* − *Z*(+*V*_*I*_ ~ −3*V*_*I*_) than the maximum value of *V*_*I*_ is defined as the asymmetric output period. The reason for the division between symmetrical and asymmetrical output sections is related to the boost method of the output voltage of the proposed single-phase embedded Z-source DC-AC inverter. When the output voltage of embedded Z-source converters *A* and *B* with a 180° phase difference is in the symmetric output region, the output voltage (*v*_*RO*_) of the proposed inverter can be in the buck region of Figure 4. But, if the negative half-cycle voltage with the gain of the symmetric and asymmetric output region is generated in the positive half-cycle, the proposed inverter can generate output of voltage (*v*_*RO*_) in the entire period of the Buck and Boost. So, when using one of the embedded Z-source converters *A* and *B*, although the output voltage is limited by the DC input voltage, the proposed single embedded Z-source inverter reverses converters *A* and *B* and using the output period of the symmetric and the asymmetric regions has the same performance as the conventional single-phase full-bridge inverter without being limited by the DC input voltage.

**Figure 5:**
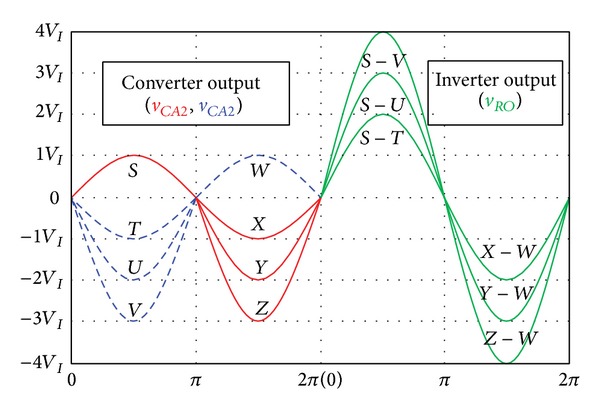
Boost method of embedded Z-source DC-AC Inverter.

Figure 5 shows the relationship of the output capacitor voltage of the embedded Z-source converters *A* and *B* and the sinusoidal waveform for the load (*R*_*O*_). *S* and *W* of the output capacitor voltage (*S*, *X*, *Y*, *Z*) of converter *A* and the output capacitor voltage (*T*, *U*, *V*, *W*) of converter *B* are the positive half-cycle voltages, which are the same as the input voltage (*V*_*I*_) of each converter, and *X*, *Y*, *Z* and *T*, *U*, *V* are the negative half-cycle voltage for one, two, and three times the input voltage (*V*_*I*_).

If the positive and negative half-cycle voltage of the converters *A* and *B* are equal to the input voltage (*V*_*I*_), the output voltage of the proposed inverter is expressed as (*S* − *T*), which is the difference between the positive half-cycle *S* of converter *A* and negative half-cycle *T* of the converter *B* and (*X* − *W*), which is the difference between the negative half-cycle *X* of converter *A* and positive half-cycle *W* of converter *B*:

(4) +vRO-Peak=Ssinπ2−(−Tsin3π2)=+2VIπ2,−vRO-Peak=−Xsin3π2−Wsinπ2=−2VI3π2,

(5) +vRO-Peak=Ssinπ2−(−3Vsin3π2)=+4VIπ2,−vRO-Peak=−3Zsin3π2−Wsinπ2=−4VI3π2.

According to (4), the output voltage (*v*_*RO*_) for the load is ±2*V*_*I*_. If an asymmetrical voltage is generated, where the output voltage of the converters *A* and *B* has the same positive half-cycle voltage magnitude as the input voltage and the same negative half-cycle voltage as three times the magnitude of the input voltage, the output voltage (*v*_*RO*_) of the proposed single embedded Z-source inverter generates the AC output voltage of ±4*V*_*I*_ by the difference between *S* − *Z* and *V* − *W*, as in (5).

### 2.3. PWM Control Method

Generally, the sinusoidal pulse width modulation (SPWM) of the single phase full-bridge inverter is based on the comparison of a triangular carrier signal with a reference sinusoidal signal. But, in the case of the proposed embedded Z-source DC-AC inverter, because the two Z-source embedded converters *A* and *B* are used, a reference waveform is needed. The output capacitor voltage (*v*_*CB*2_) of converter *B* is the same as that of converter *A*, except for the phase difference. If the positive and negative half-cycle of converter *A* have symmetric output with the maximum value of the input voltage *V*_*I*_, the relationship is expressed by (6) [6, 7]:

(6) $$\begin{array}{c} v_{CA2}=\frac{2D-1}{D}V_{I}=GV_{I}=\sin\omega t, \end{array}$$

(7) $$\begin{array}{c} G=\frac{v_{CA2}}{V_{I}}=\frac{V_{I}\sin wt}{V_{I}}=\sin\omega t. \end{array}$$

**Figure 6:**
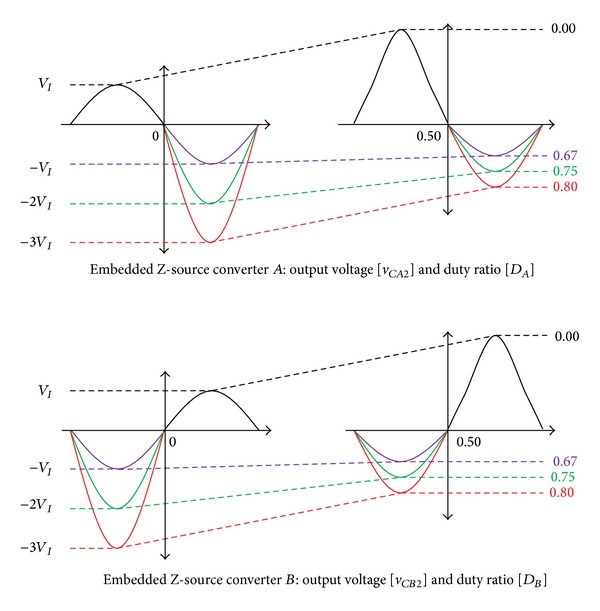
Relationship of duty ratio (*D*_*A*_, *D*_*B*_) and output voltages (*v*_*CA*2_, *v*_*CB*2_) of embedded Z-source converter *A*, *B*.

If (6) is defined by the voltage gain (*G*), it is equal to (7). Equation (8) is calculated by (6) and (7), and the short-circuit ratio (*D*) of converter *A* is equal to (9).  Figure 6 shows the relationship between the capacitor and the duty ratio (*D*) of the converter *A*, *B* for the one cycle:

(8) $$\begin{array}{c} G=\frac{2D-1}{D}=\sin wt \end{array}$$

(9) $$\begin{array}{c} D=\frac{1}{2-G}=\frac{1}{2-\sin wt}. \end{array}$$

**Figure 7:**
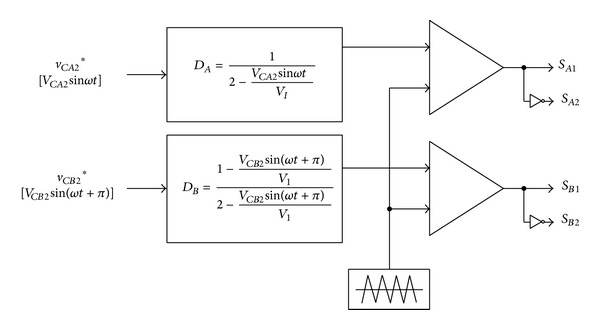
Modified SPWM controller.

Figure 6 shows the output capacitor voltage *v*_*CA*2_ of converter *A* by the short-circuit ratio (*D*_*A*_). The *D*_*A*_ curve to generate a positive half-cycle voltage shows a distorted sinusoidal waveform, and the *D*_*A*_ curve to generate the negative half-cycle voltage shows a negative voltage that becomes higher while approaching *D* = 1.0. As shown in Figure 6, if *D*_*A*_ in the negative half-cycle (*π* − 2*π*) is 0.67, a symmetric voltage with the same magnitude (−*V*_*I*_) as *V*_*I*_ is generated.  Figure 7 shows the control method of the modified SPWM.

The short-circuit ratios (*D*_*A*_, *D*_*B*_) are calculated by the order voltage (*v*_*CA*2_*, *v*_*CB*2_*) of converters *A* and *B* with a 180° phase difference and the input voltage (*V*_*I*_), and the gate signal is generated by comparison of the short-circuit ratio (*D*_*A*_, *D*_*B*_) and a chopping waveform.

## 3. Experimental Result

**Figure 8:**
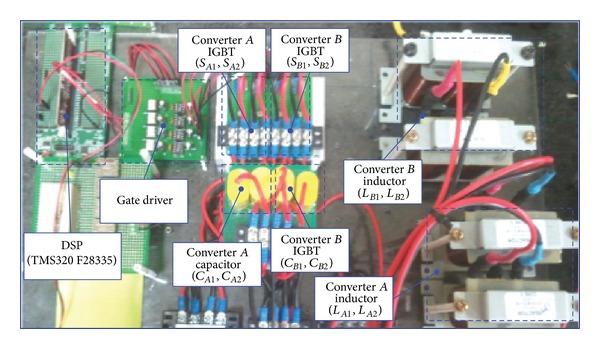
The experiment system.

To verify the proposed inverter, an experiment for the symmetric output and asymmetric output of converters *A* and *B* was performed with a DC input voltage of 38 V and a load of 100 Ω.  Figure 8 shows the experiment system. The microprocessor of the control board is a DSP (TMS320 F28335) from Texas Instruments Inc.

**Figure 9:**
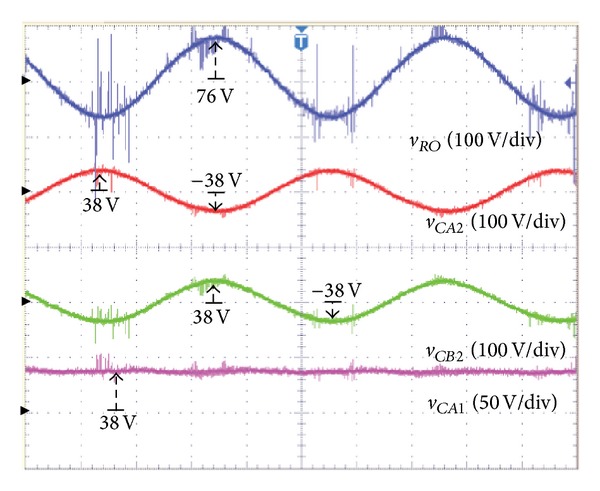
In case of symmetric output-output voltage (*v*_*RO*_) of the proposed inverter, output capacitor voltage (*v*_*CA*2_, *v*_*CB*2_) of converter *A*, *B* and input capacitor voltage (*v*_*CA*1_) of converter *A*.

**Figure 10:**
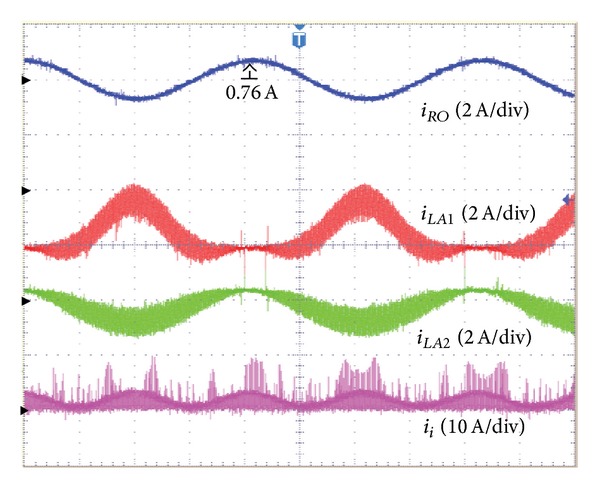
In case of asymmetric output-output current (*i*_*RO*_) of the proposed inverter, inductor current (*i*_*LA*1_, *i*_*LA*2_) of converter *A* and input current (*i*_*i*_).

An AC load with a resistance of 100 Ω was used.  Table 1 shows the system parameters, while Figures 9 and 10 show the voltage and the current of the proposed inverter for the symmetrical output.

Figure 9 shows the output voltage (*v*_*RO*_) of the proposed inverter, the output capacitor voltage (*v*_*CA*2_, *v*_*CB*2_) of converters *A* and *B*, and the input capacitor voltage (*V*_*CA*1_) of converter *A*. As shown in Figure 9, the maximum voltages of the positive and negative half-cycle are 38 V and −38 V, respectively, and the inverter generates an AC output voltage *v*_*RO*_ = 53*V*_RMS_ (76 V) with a sinusoidal waveform by the difference of this voltage.

In this case, the voltage of the capacitor has a little noise, but the same 38 V output as the input voltage *V*_*I*_ is verified.  Figure 10 shows the waveform for the current of the proposed inverter in the case of the symmetrical output. As shown in Figure 10, the output current (*i*_*RO*_) has a sinusoidal waveform.

**Figure 11:**
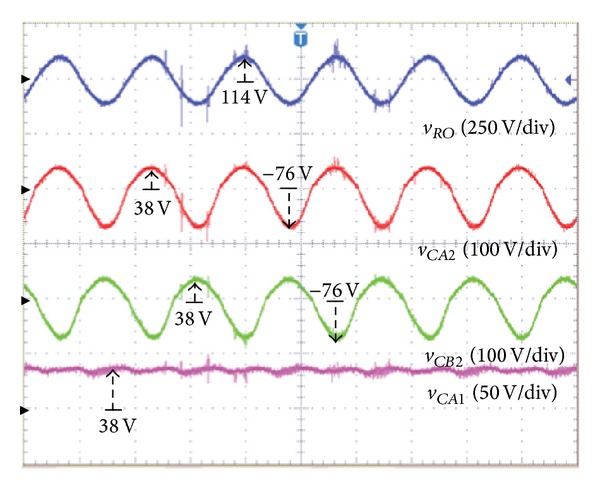
In case of asymmetric output-output voltage (*v*_*RO*_) of the proposed inverter, output capacitor voltage (*v*_*CA*2_, *v*_*CB*2_) of converter *A*, *B* and input capacitor voltage (*v*_*CA*1_) of converter *A*.

**Figure 12:**
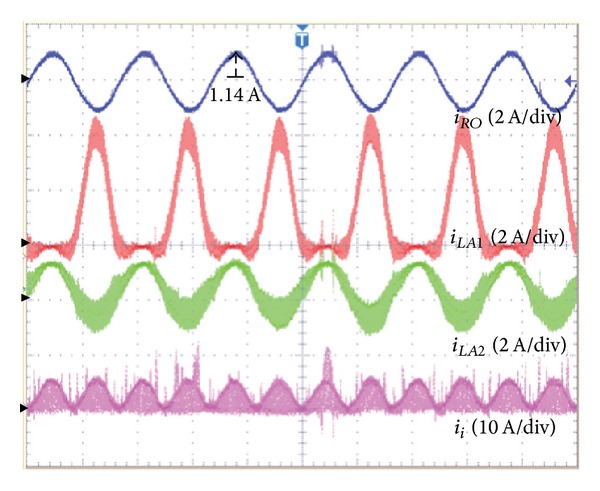
In case of symmetric output-output current (*i*_*RO*_) of the proposed inverter, inductor current (*i*_*LA*1_, *i*_*LA*2_) of converter *A* and input current (*i*_*i*_).

Figures 11 and 12 show the asymmetric output for *S* − *U* and *Y* − *W* in Figure 5. As shown in the symmetrical output, the maximum voltages of the positive half-cycle for converters *A* and *B* are the same value of 38 V as the input voltage *V*_*I*_, and the maximum voltage of the negative half-cycle is verified as −76 V, which is two times the magnitude of the input voltage *V*_*I*_. The proposed inverter generates a sine wave of 80*V*_RMS_ (114 V) by the difference between the output capacitor voltage of converters *A* and *B*. It is verified that the asymmetrical output is higher than the symmetrical output. In this situation, the capacitor voltage (*V*_*CA*1_) is the same as the input voltage (38 V).

Figure 12 shows the current waveform in case of asymmetric output. The output AC current (*i*_*RO*_) of the proposed inverter is the sinusoidal waveform, and the current of two inductors (*L*_*A*1_, *L*_*A*2_) for the converter *A* is increased compared to the symmetrical output.

**Figure 13:**
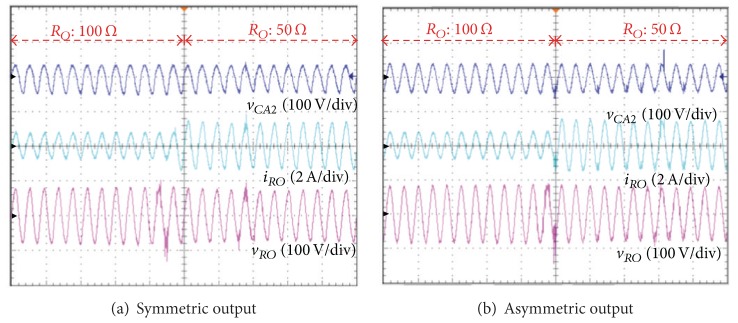
Load change characteristics of the symmetric and asymmetric output control-output capacitor voltage (*v*_*CA*2_) of converter A, output current (*i*_*RO*_), and output voltage (*v*_*RO*_) of the proposed inverter.

Figure 13 shows the experimental results for characteristics of transient state when the load is reduced suddenly to 50 Ω from 100 Ω. Load current (*v*) in 100 Ω is 0.5*A*_RMS_, and when the load is reduced to 50 Ω, load current (*i*_*RO*_) is increased to 0.85*A*_RMS_. In the case of the symmetrical and asymmetrical output control, although the current is increased by the load (*R*_*O*_) change, the output voltage (*v*_*RO*_) and current (*i*_*RO*_) are verified to maintain normally the sinusoidal waveforms.

In this situation, the capacitor voltage (*v*_*CA*1_) is the same as the input voltage (38 V). Figure 12 shows the current waveform in the case of asymmetric output. The output AC current (*i*_*RO*_) of the proposed inverter is a sinusoidal waveform, and the current of the two inductors (*L*_*A*1_, *L*_*A*2_) for the converter *A* is increased compared to the symmetrical output.

Figure 13 shows the experimental results for characteristics of the transient state when the load is reduced suddenly to 50 Ω from 100 Ω. The load current (*i*_*RO*_) for 100 Ω is 0.5*A*_RMS_, and when the load is reduced to 50 Ω, the load current (*i*_*RO*_) is increased to 0.85*A*_RMS_. In the symmetrical and asymmetrical output control, although the current is increased by the load (*R*_*O*_) change, the output voltage (*v*_*RO*_) and current (*i*_*RO*_) are maintained normally in the sinusoidal waveforms.

**Figure 14:**
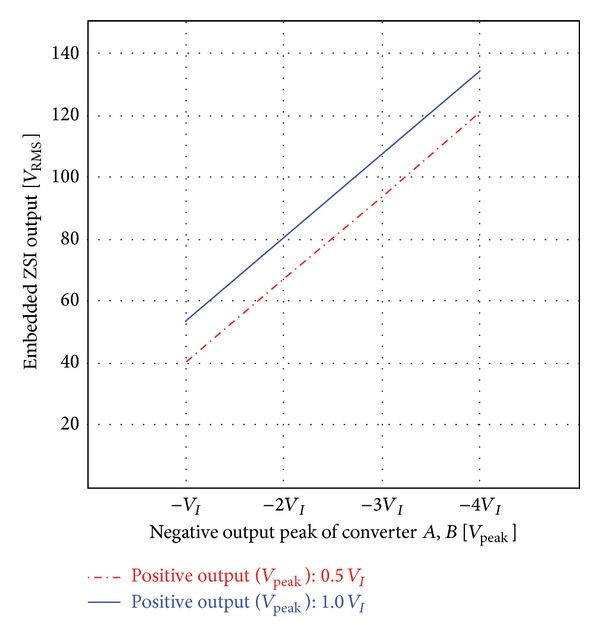
Output voltage (rms) of embedded Z-source DC-AC inverter according to negative half-cycle (0.5*V*_*I*_, 1.0*V*_*I*_) of voltage converter *A*, *B*.

Figure 14 shows the graph for the output voltage (rms) of the single embedded Z-source DC-AC by the output voltage of the proposed converters *A* and *B*. The solid line shows the output voltage of the embedded Z-source DC-AC inverter when the voltage of the negative half-cycle increases to multiples of the input voltage in conditions of the same positive half-cycle voltage.

As shown in Figure 11, if the voltage of the negative half-cycle is two times (−76 V) the DC input voltage, the output voltage (rms) of the embedded Z-source DC-AC inverter is about 80*V*_RMS_. The dotted line is the output by the voltage of the negative half-cycle when the voltage of the positive half-cycle for converters *A* and *B* is half (19 V) of the input voltage.

At the maximum voltage of the positive half-cycle for the symmetrical output control and asymmetrical output control, the minimum voltage of the negative half-cycle for the symmetrical output control is −38 V, and the minimum voltage of the negative half-cycle for asymmetrical output control is −76 V (see Table 2).

In these conditions, the output voltage of the inverter is 53*V*_RMS_ for symmetrical output control, but the output voltage of the inverter is 80*V*_RMS_ for the asymmetrical output control. So, it is verified that the asymmetrical output control is 1.5 times higher than the symmetrical output control. The efficiency of the symmetrical output is about 2% higher than that of the asymmetrical output, and it is verified that the efficiency of the proposed system is over 95%.

## 4. Conclusion

An embedded Z-source DC-AC inverter using an asymmetrical output capacitor voltage of the embedded Z-source converter was proposed. The proposed single-phase inverter generates boosted AC output voltage by combining the generated asymmetrical voltages from two converters. In comparison with the inverter using the conventional DC-DC converter, the proposed method can generate the same output voltage with low converter voltages, and the voltage stress of the system is reduced.

To verify the proposed method, a controlled single embedded Z-source inverter was made using a DSP. With an input voltage of 38 V, an experiment was conducted by symmetrical and asymmetrical output control for the output capacitor voltages of converters *A* and *B*. It was shown that the proposed inverter generated sinusoidal outputs, and it was verified that the asymmetrical output control generated higher AC voltage than the asymmetrical output control. Lastly, it was verified that the overall efficiency of the proposed inverter was over 95%.

## Figures and Tables

**Table 1:** System parameter.

Output power	500 W
Input DC voltage (*V*_*I*_)	38 V
Output AC voltage (*v*_*o*_)	53 V_RMS_~85 V_RMS_/60 Hz
Switching frequency (*f*_sw_)	20 kHz
Inductor (*L*_*A*1_, *L*_*A*2_, *L*_*B*1_, *L*_*B*2_)	1,000 *μ*H/10 A
Capacitor (*C*_*A*1_, *C*_*B*1_, *C*_*A*2_, *C*_*B*2_)	10 uF/250 V
Ac load (*R*_*o*_)	100 Ω, 50 Ω

**Table 2:** Experimental comparison of the proposed system according to the symmetric and asymmetric output control.

	Symmetrical output control	Asymmetrical output control
Output voltage of inverter (*v*_*RO*_)	53 V_RMS_	80 V_RMS_
Maximum voltage of positive half-cycle of converter (*v*_*CA*2_, *v*_*CB*2_)	38 V	38 V
Minimum voltage of negative half-cycle of converter (*v*_*CA*2_, *v*_*CB*2_)	−38 V	−76 V
Input current of inverter (*I*_*i*_)	0.76 A	1.77 A
Output current of inverter (*i*_*RO*_)	0.53 A_RMS_	0.80 A_RMS_
Efficiency (*η*)	97.1%	95.1%
